# Adherence to World Cancer Research Fund and the American Institute for Cancer Research cancer prevention recommendations and reduction in breast cancer risk: results of a large-scale case–control study in Morocco

**DOI:** 10.1017/S0007114526106527

**Published:** 2026-06-14

**Authors:** Najoua Lamchabbek, Siham Mrah, Najia Mane, Chaimaa Elattabi, Imad El Badisy, Abdellatif Bour, Fatima Zahra El M’rabet, Adil Najdi, Nawfel Mellas, Karima Bendahou, Saber Boutayeb, Lahcen Belyamani, Elodie Faure, Inge Huybrechts, Mohamed Khalis

**Affiliations:** 1 Mohammed VI International School of Public Health, Mohammed VI University of Sciences and Health, Casablanca, Morocco; 2 Department of Public Health and Clinical Research, Mohammed VI Center for Research and Innovation, Rabat, Morocco; 3 Laboratory Research of Cancer and Chronic Diseases, Faculty of Medicine and Pharmacy of Tangier, Abdelmalek Essaadi University, Tetouan 93000, Morocco; 4 Department of Epidemiology, Faculty of Medicine and Pharmacy of Fez, Sidi Mohamed Ben Abdellah University, Fez, Morocco; 5 Laboratory of Biology and Health, Department of Biology, Faculty of Sciences, University Ibn Tofail, Kenitra, Morocco; 6 Department of Oncology, Hassan II University Hospital of Fez, Fez, Morocco; 7 Department of Epidemiology, Faculty of Medicine and Pharmacy of Casablanca, Hassan II University of Casablanca, Casablanca, Morocco; 8 Department of Medical Oncology, National Institute of Oncology, Rabat, Morocco; 9 Université Paris-Saclay, UVSQ, Inserm, Gustave Roussy, CESP, 94805 Villejuif, France; 10 International Agency for Research on Cancer, World Health Organization, Lyon, France; 11 Higher Institute of Nursing Professions and Health Techniques, Ministry of Health and Social Protection, Rabat, Morocco

**Keywords:** WCRF/AICR recommendations, Observational study, Breast cancer, Lifestyle factors

## Abstract

Limited evidence exists on the combined effects of lifestyle factors on breast cancer (BC) risk, particularly in developing countries. This study aimed to investigate the association between adherence to the World Cancer Research Fund and the American Institute for Cancer Research (WCRF/AICR) cancer prevention recommendations and BC risk among Moroccan women. We conducted a large case–control study including 1,400 cases and 1,400 matched controls (by age and place of residence) between 2019 and 2023. A structured general questionnaire and a validated Food Frequency Questionnaire were used for data collection. Adherence to cancer prevention recommendations was assessed using a score ranging from 0 to 7, comprising seven components covering dietary patterns, physical activity, healthy weight, and breastfeeding. Multivariable logistic regression models were used to estimate OR and 95 % CI, accounting for potential confounding variables. For each one-point increase in the WCRF/AICR adherence score, the odds of BC decreased by 67 % overall (OR = 0·33; 95 % CI: 0·29–0·37). This inverse association was consistent among both premenopausal women (OR = 0·29; 95 % CI: 0·24–0·35) and postmenopausal women (OR = 0·35; 95 % CI: 0·30–0·41). Analysis of individual recommendations indicated that physical activity, maintaining a healthy weight, breastfeeding, consuming a plant-rich diet, and limiting the intake of fast and other processed foods were the main drivers of the observed inverse association with BC. In conclusion, greater adherence to the WCRF/AICR recommendations is associated with a reduced risk of breast cancer in Morocco. Prevention strategies should incorporate comprehensive interventions targeting multiple lifestyle factors.

Breast cancer (BC) is globally the most common cancer diagnosed among women and a leading cause of mortality^(1)^. BC represents 23·8 % of total female cancer cases worldwide, accounting for more than 2 million new cases and over 666 thousand deaths according to the latest figures published in the Global Cancer Observatory (2022)^(2)^. In recent decades, a concerning and rapid rise of BC incidence and mortality has been observed, especially in low-and middle-income countries (LMIC), where lifestyle transitions driven by globalisation and urbanisation substantially contributed to this rise^(3,4)^. Morocco, one of these LMIC that face challenges in managing the burden of BC, which, according to the recent statistics from the Cancer Registry of Casablanca, BC is the first cancer site diagnosed among women, accounting for 38·1 % of all cancer cases with an age-standardised rate of 45,5 /100·000 women^(5)^.

Over the years, growing evidence has highlighted the role of modifiable lifestyle factors, such as obesity, physical activity, alcohol consumption, smoking and diet in cancer prevention including BC^(6–9)^. These lifestyle factors often interact with each other, making it challenging to estimate the separate associations between individual lifestyle factors and cancer outcomes^(10)^. Subsequently, researchers shifted their focus to lifestyle indexes that incorporate those modifiable risk factors, primarily developed based on established recommendations aimed at preventing chronic diseases such as cancer^(11,12)^.

Based on the World Cancer Research Fund and the American Institute for Cancer Research (WCRF/AICR) continuous update project, the WCRF/AICR have published in 2007 eight evidence-based recommendation related to body fat, physical activity and diet to promote cancer prevention^(13)^. In 2018, WCRF/AICR have published ten updated guidelines for cancer prevention based on the latest scientific evidence^(14)^. Since the publication of the 2007 WCRF/AICR guidelines, numerous studies have assessed the relationship between adherence to these guidelines and cancer risk, including BC, most of these studies reported an inverse association with BC risk^(15)^. These studies have often used various scoring algorithms to assess adherence to the recommendations, what complicates the comparability of findings across different studies. To address this issue, just after the publication of the updated WCRF/AICR guidelines in 2018, a standardised scoring algorithm was developed to assess adherence to the updated recommendations and enhance comparability between studies, through a collaborative effort involving international experts including researchers from USA National Cancer Institute (NCI) and WCRF/AICR Continuous Update Project Expert Panel^(16)^.

Studies examining adherence to the updated recommendations have consistently reported an inverse association with BC risk^(14)^. However, only eleven primary studies were published so far and most were conducted in high-income countries (*n* 10), which differ from LMIC in terms of dietary patterns and overall lifestyle habits^(17–27)^. These differences highlight the need to assess the adherence to these recommendations and its association with BC risk in non-Western countries to ensure their global applicability.

Morocco, as an LMIC with distinct environmental, cultural and lifestyle characteristics, is also facing a concerning increase in BC cases^(5)^. Subsequently, Morocco provides a relevant sitting for investigating the role of modifiable lifestyle factors on BC risk. Examining these factors within this context may provide valuable insights and address critical knowledge gaps regarding adherence to these recommendations among populations with similar socioeconomic and cultural profiles.

To the best of the authors’ knowledge, no studies have so far examined the association between WCRF/AICR score and BC risk among Moroccan women. The present study is the first large case–control investigation to assess whether higher adherence to these cancer prevention guidelines is associated with a reduced risk of BC in Morocco. Additionally, the study examines which specific recommendations are driving the association with a BC risk.

## Materials and methods

### Study design and setting

The database was derived from the BREAST Morocco study, a multicentre case–control study conducted across various hospital centres in multiple cities in Morocco (Ibn Rochd University Hospital in Casablanca, Hassan II University Hospital in Fez, Al Hoceima Oncology Centre, Sheikh Zayed Al Nahyan Oncology Hospital in Tangier and Al Hassani Provincial Hospital in Nador) between December 2019 and August 2023. The study involves a large sample size of 2800 participants, including 1400 cases and 1400 controls, matched by age (±5 years) and place of residence (urban or rural). The sample size for this case–control study had sufficient power to detect an OR of 1·5 or greater, of 80 % and a two-sided significance level of 5 % (*α* = 0·05).

### Study population

All included BC cases were histologically confirmed as primary *in situ* or invasive diagnoses before undergoing any surgical intervention or cancer treatment, such as chemotherapy or radiotherapy. These cases were recruited from the oncology centre at the study sites.

Control participants were recruited within a maximum of 2 months of their matched case. They were randomly selected from the same hospital as the cases, specifically among healthy women attending with patients at the outpatient consultation centres for conditions unrelated to cancer, and with no current or prior history of cancer.

Eligibility criteria for both cases and controls included not being pregnant or lactating at the time of the study and having the ability to provide informed consent.

### Data collections and assessment

During enrolment in this multicentre case–control study, six trained interviewers filled out face-to-face questionnaires and carried out all measurements with participants. The general questionnaire included socio-demographic data, such as age, education level, marital status and employment history. Also, it collected information about family cancer history and detailed reproductive health factors, such as age at menarche, parity, age at first full-term pregnancy, breast-feeding practices and menopausal status. Additionally, this questionnaire recorded participants’ hormone use, exposure to smoking, alcohol consumption and physical activity. More information about this study and the questionnaires was detailed previously^(28)^.

#### Socio-economic status, anthropometric and physical activity assessment

The participants’ socio-economic position was evaluated using a validated wealth score constructed based on various household assets. Morocco was one of the nations included in the development and validation of this socio-economic assessment approach, making it particularly well-suited for studies conducted in this context^(29)^.

To assess various aspects of body composition and fat distribution, a set of anthropometric measurements was collected such as weight, height, seated height, hip circumference and waist circumference, each measured using standardised and validated protocols to ensure reliability and consistency^(30)^. Height was measured using a stadiometer, with participants standing on a flat, uncarpeted surface. Weight was recorded using a calibrated digital scale placed on a flat, stable surface. Participants were weighed in light clothing and without shoes to obtain accurate body mass measurements. The BMI was calculated by dividing the weight (in kilograms) by the square of the height (in metres) (BMI = weight (kg)/height^2^ (m)).

Physical activity (PA) was assessed using a structured questionnaire, previously employed in a published study conducted in Morocco^(31)^, with detailed information that capture physical activity level over the past 12 months and during life stages. The questionnaire captured self-reported PA across different domains, including occupational, recreational and household activities. Participants were asked to estimate the average number of hours spent per week in low, moderate and vigorous-intensity activities from Monday to Sunday, based on a typical week that reflects their usual lifestyle last year. The interviewers provided examples of PA types to help participants distinguish between intensity levels. In addition, participants were asked to describe their PA level during earlier life stages of life (childhood, adolescence and early adulthood) and the types and frequency of habitual activities including walking, stairs clamping and occupational tasks.

This approach allowed for the categorisation of individuals into different physical activity levels for subsequent analysis.

#### Dietary intake assessment

The dietary intake of participants was assessed using a validated Food Frequency Questionnaire (FFQ). The cases groups were asked to report their dietary habits during the last year preceding their diagnosis and for the control group to report dietary habits during the year prior to the interview. This FFQ was developed based on the European Global Asthma and Allergy Network’s (GA2LEN) FFQ, which was validated and adapted to reflect Moroccan context ensuring cultural relevance and applicability^(32)^.

The FFQ used in this study comprises a comprehensive list of 255 food items, grouped into thirty-two primary food categories specific of Moroccan cuisine. These food groups capture traditional Moroccan foods accounting for the rich diversity of Moroccan culinary traditions. Participants were asked to provide detailed responses for each food item listed. Nine frequency options were available, ranging from ‘never’ to ‘six or more times per day’. Additionally, portion size for each food item consumed was specified, using traditional local household measurements such as spoons, cups, bowls and plates. To calculate daily food intake, the frequency of consumption for each food item was multiplied by the portion size. Nutrient intake data were derived using food composition tables specific to the Moroccan population^(33)^.

### Structure and operationalisation World Cancer Research Fund and the American Institute for Cancer Research recommendations score

To enhance the comparability of the present study results with other studies used the 2018 WCRF/AICR guidelines to assess BC risk factors, we structured our score in accordance with the standardised scoring methodology recently proposed by the NCI collaborative group, whenever possible^(16)^. Our adherence score incorporated one specialised (breast-feeding if you can) and seven general WCRF/AICR recommendations (maintaining a healthy weight, engaging in regular physical activity, consuming a diet rich in wholegrains, vegetables, fruits and legumes, limiting the intake of energy-dense processed foods, red and processed meat consumption and sugar-sweetened beverages). The recommendation ‘limit alcohol consumption’ was not operationalised in this study, as 98·4 % of the study population reported no alcohol consumption. Consequently, this component was excluded from the score calculation. Each of the included components of the WCRF/AICR recommendations was calculated based on the predefined criteria from the NCI standardised score, using data collected through lifestyle and dietary questionnaires as follows:

1. Maintain a healthy weight: This recommendation was operationalised using two subcomponents measured waist circumference (cm) and BMI (kg/m^2^), calculated from measured height and weight, scored in accordance with National Cancer Institute guidelines.
2. Be physically active: To reflect relative levels of physical activity within the study population and due to data limitations in precisely assessing the NCI recommended cut-offs, we used control-based tertiles to categorise participants’ levels of moderate PA (0 points for the lowest tertile, 0·5 points for the middle tertile and 1 point for the highest tertile).
3. Eat a diet rich in wholegrains, vegetables, fruits and legumes: this recommendation was measured by two subcomponents, the daily intake of fruits and vegetables and dietary fibre (g/d), and compliance was assigned in accordance with the NCI’s scoring algorithm.
4. Limit consumption of ‘fast foods’ and processed foods high in fat, refined starches or sugars: in accordance with NCI scoring algorithm, this component was assessed using the NOVA classification system(34), which classifies foods items based on their degree of processing. To ensure specificity, food items already included in other components of the score (sugar-sweetened drinks, red meats and processed meats classified as ultra-processed foods) were excluded from the original NOVA UPF variable. An adapted variable, termed adapted ultra-processed food, was created. The percentage of total daily energy intake from aUPF was calculated and categorised into tertiles based on the control group (0 point for the lowest tertile, 0·5 points for the middle tertile and 1 point for the highest tertile).
5. Limit consumption of red and processed meat: Compliance was evaluated by assessing the daily intake of red and processed meat using FFQ data, and scoring was conducted in accordance with NCI scoring algorithm.
6. Sugar-sweetened beverages: this component was assessed using a the culturally adapted FFQ. In which, participants reported their usual intake of commonly consumed sweetened drinks, and compliance was assigned in accordance with the NCI’s scoring algorithm.
7. For mothers: breastfeed your baby, if you can: Breast-feeding information was collected through a detailed reproductive history questionnaire. The average duration was calculated by dividing the reported total duration by the number of children breastfed. The NCI scoring algorithm was applied only to parous women.

Each participant received a score based on their adherence to these recommendations. Specifically, one point was assigned for full adherence, half a point for partial adherence and zero points for non-adherence. For the two recommendations (eat a diet rich in wholegrains, vegetables, fruits and legumes and maintaining healthy weight) composed of two subcomponents, each subcomponent was scored separately, and the points were averaged to reflect balanced weighting (0 for non adherence, 0·25 for partial adherence and 0·5 and for full adherence).

The operationalisation of these components following the NCI standardised scoring algorithm and detailed criteria for assessing compliance are described in Table 1.

**Table 1.** Operationalisation and distribution of the 2018 WCRF/AICR cancer prevention score components among cases and controls using the NCI standardised algorithm^*^ Table 1 long description.

				Controls (*n* 1373)		Cases (*n* 1373)		*P*
2018 WCRF/AICR cancer prevention recommendations	Variables in the study	Operationalisation of the components of the score based on the NCI-led standardised scoring algorithm^*^	Score	*n*	%	*n*	%	
1. Be a healthy weight^*^ ^,†^	BMI (kg/m^2^) derived from measured height and weight	BMI < 18·5 OR BMI ≥ 30	0	173	12·9 %	300	21·8 %	< 0·001
		25 ≤ BMI < 30	0·25	782	58·2 %	699	50·9 %	
		18·5 ≤ BMI < 25	0·5	389	28·9 %	374	27·2 %	
	Measured waist circumference (cm)	WC ≥ 88	0	886	65·5 %	1182	86·1 %	< 0·001
		80 ≤ WC < 88	0·25	237	17·5 %	111	8·1 %	
		WC < 80	0·5	229	16·9 %	80	5·8 %	
2. Be physically active	Moderate physical activity (min/week)	Lowest tertile (≤ 33·3rd percentile).	0	428	31·2 %	588	42·8 %	< 0·001
		Middle tertile (33·3rd–66·6th percentile).	0·5	423	30·8 %	428	31·2 %	
		Highest tertile (≥ 66·6th percentile).	1	521	38·0 %	357	26·0 %	
3. Eat a diet rich in wholegrains, vegetables, fruit and beans^*^ ^,†^	Fruits and vegetables items in the FFQ: tomatoes, aubergine, courgette, peppers (red, raw, yellow), cucumber, carrots, parsnip, swede, artichoke, radish, beetroot, chilli peppers, sweet corn kernels, asparagus, spinach, lettuce, fenugreek seeds, Rejla (Bakkoula), Mloukhia (Jew’s Mallow), aromatic herbs (mint, basil, parsley, coriander), leeks, mushrooms, onions, garlic, cauliflower, pumpkin, brussels sprouts, broccoli, cabbage, tomatoes stuffed with vegetables, pickle, mixed vegetables, ginger root, apples, pears, bananas, peaches, avocados, cherries, mulberries, blackcurrants, raspberries, watermelon, grapes, mangoes, apricots, nectarines, plums, pineapple, kiwi fruit, oranges, mandarins, grapefruit, lemon, fruit cocktail, canned fruits, figs, raisins, dates, and dried mixed fruit, along lemon pickles and black or green olives. Fresh orange juice, fresh pomegranate juice and other types of fresh fruit juices.	Fruits and vegetables intake < 200 g/d	0	419	30·5 %	888	64·7 %	< 0·001
		200 g/d ≤ Fruits and vegetables intake < 400 g/d	0·25	428	31·2 %	272	19·8 %	
		Fruits and vegetables intake ≥ 400 g/d	0·5	526	38·3 %	213	15·5 %	
	Fibre intake g/d calculated based on the FFQ	Dietary fibre intake < 15	0	127	9·2 %	171	12·5 %	< 0·001
		15 ≤ Dietary fibre intake < 30	0·25	262	19·1 %	328	23·9 %	
		Dietary fibre intake ≥ 30	0·5	984	71·7 %	874	63·7 %	
4. Limit consumption of ‘fast foods’ and other processed foods high in fat, refined starches or sugars^*^	The percentage of total daily energy intake from adapted ultra-processed foods (aUPF), according to the NOVA classification based on the FFQ, included the following items: Madeleine cake, date cake, croissants, Moroccan sweets (almond sweets), semolina sweets (Basboussa, Maqrout), ring doughnuts, coconut sweets, pancake rolls, honey-dipped pastries (Chabbakia, Mkharrka), jam (fruit spread), chewy sweets, fudge, toffees, cereal chewy bars, ice creams, chocolate-covered bars (with fruit, nuts or biscuits), margarine and vegetable fat, animal fat (butter), potato French fries/chips, Maakoda, pizza, ketchup, mayonnaise, and mustard sauce.	Low Adherence: High intake (≤ 33·3rd percentile).	0	457	33·3 %	815	59·4 %	< 0·001
		Moderate Adherence: Moderate intake (33·3rd–66·6th percentile).	0·5	458	33·4 %	392	28·6 %	
		High Adherence: Low intake (≤ 33·3rd percentile).	1	458	33·4 %	166	12·1 %	
5. Limit consumption of red and processed meat^*^	Red and processed meat items in the FFQ: grilled beef, fillet, cutlet, beef in tagine, ground beef, grilled lamb, lamb in tagine, ground lamb, goat, veal, camel meat, meat (rabbit, duck, pigeon, partridge) Sausage, shredded beef, shredded lamb, dried meat, processed fish, processed poultry.	Red and processed meat ≥ 500 g/week or processed meat intake ≥ 50 g/d	0	48	3·5 %	41	3·0 %	0·451
		Red and processed meat < 500 g/week; processed meat intake of 3 to < 50 g/d	0·5	–	–	–	–	
		Red and processed meat < 500 g/week of which processed meat intake < 3 g/d	1	1325	96·5 %	1332	97·0 %	
6. Limit consumption of sugar-sweetened drinks^*^	Carbonated drinks (lemonade), fruit juices, tea.	Sugary drinks intake > 250 g/d	0	587	42·8 %	685	49·9 %	< 0·001
		Sugary drinks intake ≤ 250 g/d	0·5	659	48·0 %	590	43·0 %	
		Sugary drinks intake = 0 g/d	1	127	9·2 %	97	7·1 %	
7. For mothers: breastfeed your baby, if you can^*^ ^,‡^	The average duration was calculated by dividing the reported total duration by the number of children breastfed	No breast-feeding	0	13	1·3 %	43	4·3 %	< 0·001
		> 0–6 months	0·5	35	3·5 %	70	6·9 %	
		≥ 6 months	1	964	95·3 %	897	88·8 %	
8. Limit alcohol consumption	Not calculated because 98·4 % of the study population reported no alcohol consumption.							
9. Do not rely on supplements for cancer prevention	Not assessed in this study							
10. After a cancer diagnosis: follow our recommendations, if you can	Not applicable to this population							

WCRF, World Cancer Research Fund; AICR, American Institute for Cancer Research; PA, physical activity; MET, metabolic equivalents; aUPF: adapted ultra-processed food.

*P* value; χ 2 test for qualitative variables.

* The score is based on NCI Standardised Algorithm.

† Adherence to the recommendation ‘Be a healthy weight’ is measured by two subgroups, BMI and waist circumference; adherence to the recommendation ‘Eat a diet rich in wholegrains, vegetables, fruit and beans’ is measured by two subgroups, fruit and vegetable intake and daily fibre intake.

‡ Recommendation only applied to parous women.

### Statistical analysis

#### Data handling and cleaning

To address missing data, single imputation was performed using the median for continuous variables and the mode for categorical variables, as all variables had less than 5 % missing values, ensuring consistency in the data analysis. To address bias in self-reported dietary data, we compared participants’ energy intake to their estimated energy requirements, calculated using Schofield equation that accounts for age, gender, weight, height and activity level. Participants in bottom 1 % energy intake/estimated energy requirement ratio were identified as under-reporters and excluded from the study. As a result, fifty-four participants were removed, and the final sample includes 2746 participants^(35–37)^.

The general characteristics of the population are presented using mean values for continuous variables and percentages for categorical variables. Logistic regression models were used to estimate OR of BC and the 95 % CI for each individual recommendation and for the overall WCRF/AICR score. Stratification by menopausal status (premenopausal *v*. postmenopausal) was used for both overall and for each individual recommendation. In the multivariate analyses, we adjusted for age at menarche (years), average daily caloric intake (kcal/day), physical activity (MET min/week), wealth score, educational level (illiterate, elementary /Koranic school, secondary school and high school/technical or professional school), occupation (housewife, employed and previous employed), history of oral contraceptive use (Yes, No), age at first full-term pregnancy (nulliparous, < 22 years and > 22 years), breast-feeding (never breastfeed, > 0–< 24 months, ≥ 24 month and nulliparous), age at menopause (premenopausal, < 50 years, > 50 years), family history of BC (Yes, No) and BMI (< 25 kg/m^2^, 25–29 kg/m^2^ and ≥ 30 kg/m^2^) (unless the variable was part of the recommendation evaluated). Additionally, in the stratified analysis by menopausal status, additional adjustments for the original matching variables, age and region were included, while no adjustment was made for age at menopause.

Tertiles of adapted ultra-processed food, physical activity and WCRF/AICR score were derived from the distribution among controls. All statistical analyses were performed with SPSS version 21.0 and were conducted separately for premenopausal and postmenopausal women.

## Results

The general characteristics of the cases and controls in the study population are presented in Table 2. Compared to controls, women with BC were more likely to report a younger age at menarche, an older age at first full-term pregnancy and an older age at menopause. Use of oral contraceptives was also more frequent among cases. Furthermore, among parous women, breast-feeding practices differed markedly, with a significantly higher proportion of controls reporting breast-feeding for 24 months or more. A family history of cancer and BC was also more prevalent among cases.

**Table 2.** General characteristics of the study population by case and control groups (*n* 2746) Table 2 long description.

	Controls (1373)		Cases (1373)		*P* value^*^
	Mean	sd	Mean	sd	
Age	50·5	11·4	50·9	11·4	0·300
Age at menarche (years)	12·9	1·4	12·7	1·5	< 0·001
Age at full-term pregnancy (years)^†^	22·4	5·2	23·6	5·5	< 0·001
Age at menopause (years)^‡^	48·9	5·1	50·2	5·1	< 0·001
Wealth score	7·4	1·7	6·9	1·8	< 0·001
Moderate physical activity (min /week)	968·7	649·52	781·9	572·3	< 0·001
BMI	26·58	3·39	27·30	4·44	< 0·001
WC	94	14	102	13	< 0·001
HC	110	13	113	12	< 0·001
Energy intake (kcal/day)	2605·5	980·9	2508·0	738·8	0·003
Vegetables (g/d)	58·30	51·53	46·13	37·04	< 0·001
Fruits (g/d)	65·71	61·31	50·19	46·86	< 0·001
Unsaturated oils (g/d)	34·43	23·05	24·59	19·02	< 0·001
Nuts and seeds (g/d)	7·12	13·28	3·66	8·05	< 0·001
Whole grains (g/d)	71·84	107·97	33·60	65·52	< 0·001
Red meat (g/d)	21·92	31·07	23·38	31·53	0·224
Processed meat (g/d)	5·28	14·05	5·23	12·86	0·922
Fibre intake (g/d)	45·13	24·33	39·98	22·16	< 0·001
	*n*	%	*n*	%	
Area of residence					1
Urban	935	68·1 %	935	68·1 %	
Rural	438	31·9 %	438	31·9 %	
Marital status					0·056
Single	251	18·3 %	240	17·5 %	
Married	937	68·2 %	905	65·9 %	
Divorced	85	6·2 %	88	6·4 %	
Widowed	100	7·3 %	140	10·2 %	
Education level					< 0·001
Illiterate	644	59·4 %	790	68·3 %	
Elementary/Koranic school	292	26·9 %	300	25·9 %	
Secondary school	288	26·5 %	215	18·6 %	
High school/Technical or professional school	149	13·7 %	67	5·8 %	
Occupation					< 0·001
Housewife	1007	73·3 %	946	68·9 %	
Employed	241	17·6 %	164	11·9 %	
Previously employed	125	9·1 %	263	19·2 %	
Oral contraceptive					< 0·001
Yes	609	45·2 %	809	59·4 %	
No	738	54·8 %	553	40·6 %	
Parity					0·931
Parous	1017	74·1 %	1015	73·9 %	
Nulliparous	356	25·9 %	358	26·1 %	
Breast-feeding^†^					< 0·001
Never breastfeed	13	1·3 %	43	4·3 %	
> 0–< 24 months	146	14·4 %	281	27·8 %	
≥ 24 months	853	84·3 %	686	67·9 %	
Family history of cancer					< 0·001
Yes	263	19·2 %	414	30·2 %	
No	1110	80·8 %	959	69·8 %	
Family history of breast cancer					< 0·001
Yes	77	5·6 %	181	13·2 %	
No	1296	94·4 %	1192	86·8 %	
Alcohol					0·412
Yes	9	0·7 %	6	0·4 %	
No	1334	97·2 %	1367	99·6 %	
Smoking exposure					0·796
Yes	135	9·8 %	131	9·5 %	
No	1238	90·2 %	1242	90·5 %	

Continuous variables are presented as means (sd) and categorical variables are presented as frequencies (%).

WC, waist circumference, WC, waist circumference, HC, hip circumference.

* *P* value; χ 2 test for categorical variables ant student *t* test for continuous variables.

† Among parous women.

‡ Among postmenopausal women.

Furthermore, cases had a lower wealth score, were less likely to have attained a higher educational level or be physically active and exhibited significantly higher mean values for all anthropometric indicators, including BMI, WC and HC.

Regarding dietary intake, total energy intake was slightly lower in cases than in controls. Compared with controls, cases consumed significantly fewer vegetables, fruits, unsaturated oils, nuts and seeds, whole grains and dietary fibre. Socio-demographic characteristics were broadly comparable between groups concerning education level differed, with a higher proportion of illiterate women among cases compared with controls. Likewise, occupational status differed.

Table 3 shows the OR (CI) for BC in relation to the WCRF/AICR score, both overall and stratified by menopausal status. Compared with the lowest adherence category, a significant trend of decreasing BC risk with increasing adherence was observed in the crude model, overall (OR = 0·20 (0·14, 0·26), *P* < 0·001) in premenopausal women (OR = 0·20 (0·14, 0·26), *P* < 0·001) and in postmenopausal women (OR = 0·11 (0·10, 0·16), *P* < 0·001). After adjusting for confounding factors, this inverse trend remained significant, overall (OR = 0·14 (0·11, 0·18), *P* < 0·001) as well as in premenopausal women (OR = 0·13 (0·09, 0·19), *P* < 0·001) and in postmenopausal women (OR = 0·12 (0·08, 0·17), *P* < 0·001). Furthermore, each one-point increase in adherence to the WCRF/AICR score was inversely associated with BC risk overall (OR = 0·33; 95 % CI: 0·29, 0·37, *P* < 0·001), as well as among both premenopausal women (OR = 0·29; 95 % CI: 0·24, 0·35, *P* < 0·001) and postmenopausal women (OR = 0·35; 95 % CI: 0·30, 0·41, *P* < 0·001).

**Table 3.** Associations between the adherence to the overall 2018 WCRF/AICR cancer prevention recommendations using the NCI standardised scoring algorithm and breast cancer risk Table 3 long description.

	Adherence score^‡^	Controls		Cases		Unadjusted model		Adjusted model^*^	
		*n*	%	*n*	%	OR	95 % CI	OR	95 % CI
Overall (cases = 1373; controls = 1373)	≤ 3·75	414	30·2%	814	59·3	1		1	
	> 3·75 & ≤ 4·5	421	30·7%	386	28·1	0·47	0·39, 0·56	0·45	0·37, 0·55
	> 4·5	538	39·2%	173	12·6	0·16	0·13, 0·20	0·14	0·11, 0·18
	*P*-trend					< 0·001		< 0·001	
	1-point increment					0·4	0·35, 0·42	0·33	0·29, 0·37
	*P* value					< 0·001		< 0·001	
Premenopausal (overall *n* 1258; cases = 621; controls = 637)^†^	≤ 3·5	217	34·1%	382	61·5%	1		1	
	> 3·5 & ≤ 4·5	198	31·1%	164	26·4%	0·47	0·36, 0·61	0·41	0·30, 0·56
	> 4·5	222	34·9%	75	12·1%	0·20	0·14, 0·26	0·13	0·09, 0·19
	*P*-trend					< 0·001		< 0·001	
	1-point increment					0·41	0·35, 0·47	0·29	0·24, 0·35
	*P* value					< 0·001		< 0·001	
Postmenopausal (overall *n* 1485; cases = 751; controls = 734)^†^	≤ 3·75	247	33·7%	522	69·5%	1		1	
	> 3·75 & ≤ 4·5	250	34·1%	173	23%	0·33	0·26, 0·42	0·35	0·27, 0·45
	> 4·5	237	32·3%	56	7·5%	0·11	0·10, 0·16	0·12	0·08, 0·17
	*P*-trend					< 0·001		< 0·001	
	1-point increment					0·36	0·31, 0·41	0·35	0·30, 0·41
	*P* value					< 0·001		< 0·001	

Unadjusted model: Crude OR.

* Adjusted model: OR adjusted for age at menarche (years), average daily caloric intake (kcal/day), wealth score (continuous), educational level (illiterate, elementary /Koranic school, secondary school, high school /technical or professional school), occupation (housewife, employed, previous employed), history of oral contraceptive use (Yes, No), age at first full-term pregnancy (nulliparous, < 22 years,> 22 years), age at menopause (premenopausal, < 50 years, > 50 years), family history of breast cancer (Yes, No).

† Additional adjustments were made for the original matching variables (age and region), with no adjustment for age at menopause in analyses stratified by menopausal status.

‡ Tertile cut-off points were defined according to the distribution of the WCRF score among controls.

Table 4 presents the results of the association between BC risk and adherence to specific recommendations from the 2018 WCRF/AICR cancer prevention guidelines, adjusted for potential confounders. Adherence to the WCRF/AICR recommendation on maintaining a healthy weight, assessed by BMI and waist circumference, was associated with a significantly reduced BC risk. Specifically, waist circumference showed a strong inverse relationship with overall (OR = 0·18 (0·12, 0·28), *P* < 0·001) and both premenopausal (OR = 0·12 (0·06, 0·25), *P* < 0·001) and postmenopausal women (OR = 0·25 (0·15, 0·44), *P* < 0·001). For BMI, a significant inverse trend was observed only among postmenopausal women (OR = 0·60 (0·40, 0·89), *P* = 0·018), whereas no significant association was observed in both overall and premenopausal women.

**Table 4.** Associations between the adherence to individual recommendations of the 2018 WCRF/AICR cancer prevention recommendation, using the NCI standardised score and breast cancer risk Table 4 long description.

WCRF/AICR cancer prevention recommendation	Score	Overall		Premenopausal		Postmenopausal	
		(cases = 1373; controls = 1373)		(cases = 621; controls = 637)		(cases = 751; controls = 734)	
		OR^*^	95 % CI	OR^*^	95 % CI	OR^*^	95 % CI
Be a healthy weight (1) BMI	0	1		1		1	
	0·25	0·57	0·43, 0·74	0·66	0·42, 1·03	0·52	0·37, 0·74
	0·5	0·72	0·52, 0·98	0·92	0·55, 1·54	0·60	0·40, 0·89
	*P*-trend	0·072		0· 972		0·018	
Be a healthy weight (1) Waist circumference	0	1		1		1	
	0·25	0·34	0·24, 0·49	0·33	0·19, 0·56	0·34	0·21, 0·57
	0·5	0·18	0·12, 0·28	0·12	0·06, 0·25	0·25	0·15, 0·44
	*P*-trend	< 0·001		< 0·001		< 0·001	
Be physically active	0	1		1		1	
	0·5	0·48	0·38, 0·61	0·46	0·31, 0·68	0·53	0·38, 0·73
	1	0·34	0·26, 0·44	0·27	0·18, 0·41	0·43	0·31, 0·61
	*P*-trend	< 0·001		< 0·001		< 0·001	
Eat a diet rich in wholegrains, vegetables, fruit and beans (2) Fruit and vegetable intake	0	1		1		1	
	0·25	0·26	0·20, 0·35	0·15	0·09, 0·24	0·33	0·23, 0·46
	0·5	0·12	0·084, 0·16	0·07	0·04, 0·12	0·14	0·10, 0·22
	*P*-trend	< 0·001		< 0·001		< 0·001	
Eat a diet rich in wholegrains, vegetables, fruit and beans (2) fibre intake	0	1		1		1	
	0·25	0·84	0·57, 1·24	0·89	0·46, 1·72	0·77	0·48, 1·25
	0·5	0·63	0·43, 0·91	0·63	0·34, 1·19	0·59	0·38, 0·94
	*P*-trend	0·004		0·073		0·016	
Limit consumption of ‘fast foods’ and other processed foods high in fat, refined starches or sugars (aUPF)	0	1		1		1	
	0·5	0·34	0·26, 0·45	0·69	0·48, 0·99	0·36	0·26, 0·51
	1	0·13	0·10, 0·18	0·94	0·46, 1·90	0·14	0·10, 0·21
	*P*-trend	< 0·001		< 0·001		< 0·001	
Limit red and processed meat	0	1		1		1	
	1	1·43	0·70, 2·13	1·39	0·53, 3·66	1·51	0·61, 3·77
	*P*-trend	0·280		0·50		0·377	
Limit sugary drinks	0	1		1		1	
	0·5	0·79	0·63, 0·99	0·69	0·48, 0·99	0·89	0·66, 1·18
	1	0·77	0·51, 1·16	0·94	0·46, 1·90	0·78	0·47, 1·29
	*P*-trend	0·043		0·183		0·263	
Breastfeed your baby if you can	0	1		1		1	
	0·5	0·65	0·27, 1·58	0·30	0·07, 1·27	0·34	0·10, 1·13
	1	0·20	0·09, 0·43	0·21	0·06, 0·69	0·15	0·05, 0·42
	*P*-trend	< 0·001		< 0·001		< 0·001	

WCRF/AICR, World Cancer Research Fund and the American Institute for Cancer Research; aUPF, adapted ultra-processed food according to NOVA classification.

All values are OR (95 % CI), and 0 indicates the lowest adherence to the specific recommendation.

* Adjusted for age at menarche (years), average daily caloric intake (kcal/day), physical activity (MET min/week), wealth score, educational level (illiterate, elementary /Koranic school, secondary school, high school /Technical or professional school),occupation(housewife, employed and previous Employed), history of oral contraceptive use (Yes, No), age at first full-term pregnancy (nulliparous, < 22 years,> 22 years), breastfeed infants (never breastfeed, > 0–< 24 months, ≥ 24 month, missing), age at menopause (Premenopausal, < 50 years, > 50 years), family history of breast cancer (yes, no) and BMI (< 25 kg/m^2^, 25–29 kg/m^2^, ≥ 30 kg/m^2^). Unless the variable was part of the recommendation evaluated, additional adjustments were made for the original matching variables (age and region), with no adjustment for age at menopause in stratified analyses by menopausal status.

(1) Adherence to the recommendation ‘Be a healthy weight’ is measured by two subgroups, BMI and waist circumference.

(2) Adherence to the recommendation ‘Eat a diet rich in wholegrains, vegetables, fruit and beans’ is measured by two subgroups, fruit and vegetable intake and daily fibre intake.

For dietary habit-related recommendations, significant trends of reduced BC risk were observed with increased fruit and vegetable intake, both in the overall group (OR = 0·12 (0·084, 0·16), *P* < 0·001), as well as in premenopausal women (OR = 0·07 (0·04, 0·12), *P* < 0·001) and postmenopausal women (OR = 0·14 (0·10, 0·22), *P* < 0·001). Similarly, increased fibre intake was associated with reduced risk overall (OR = 0·63 (0·43, 0·91), *P* < 0·001) and in postmenopausal women (OR = 0·60 (0·38, 0·96), *P* = 0·016). Limiting adapted ultra-processed food also showed significant trends of reduced risk both overall (OR = 0·13 (0·10, 0·18), *P* < 0·001) in postmenopausal women (OR = 0·14 (0·10, 0·21), *P* < 0·001). However, no significant trend in risk emerged for adherence to recommendations on meat and processed meat consumption or sugary drinks.

Adherence to physical activity recommendations was also associated with reduced BC risk, with significant trends, overall (OR = 0·34 (0·26, 0·44), *P* < 0·001), in premenopausal women (OR = 0·27 (0·18, 0·41), *P* < 0·001) and postmenopausal women (OR = 0·43 (0·31, 0·61), *P* < 0·001). Similarly, adherence to breast-feeding recommendation was associated with a significant reduction in BC risk overall (OR = 0·20 (0·09, 0·43), *P* < 0·001), as well as in premenopausal women (OR = 0·21 (0·06 0·69), *P* < 0·001) and postmenopausal women (OR = 0·15 (0·05, 0·42), < 0·001).

## Discussion

This large-scale case–control study, the first in Morocco and among the pioneering investigations in LMIC to examine the relationship between adherence to the 2018 WCRF/AICR Guidelines and BC risk, suggests a strong inverse association between higher adherence to these guidelines and BC risk, both in the overall population and among premenopausal and postmenopausal women.

A particularly noteworthy aspect of our findings is the strength of the association between adherence to the WCRF/AICR recommendations and BC risk. The 67 % risk reduction observed for each one-point increment in our study is substantially greater than the more modest 10–11 % risk reduction reported in meta-analyses of studies from predominantly HIC^(15,38)^. This discrepancy needs careful consideration and highlights the importance of interpreting our results within the distinct context of our study population.

The existing body of evidence on the 2018 recommendations is still developing, with only a limited studies available. To date, only eleven primary studies have examined adherence to the updated 2018 recommendations in relation to BC risk, and the vast majority of these were cohort studies conducted in HIC^(17–27)^. These populations differ markedly from LMIC like Morocco in their dietary patterns, lifestyle behaviours, socio-economic contexts and stages of nutritional transition^(39,40)^. Therefore, direct comparisons should be interpreted with caution, as baseline risk profiles and environmental exposures may vary substantially between settings.

Additionally, several factors may help contextualise the strong association observed in our study. As a first consideration, we must acknowledge the potential for selection bias, a common limitation of case–control studies^(41)^. The selection of controls in our study was hospital based, which may have resulted in a comparison group that is more health conscious than the general population, which may have potentially led to an overestimation of the true magnitude of the association and partly explaining the stronger association observed. Indeed, previous meta-analytic evidence has suggested that case–control designs tend to report stronger associations than prospective cohort studies in this area of research^(14)^.

Beyond selection bias, the distinct epidemiological and nutritional context of Morocco, a nation undergoing a rapid nutrition transition, likely plays a significant role. Unlike in many HIC where dietary patterns have become more homogeneous^(42)^. Morocco is characterised by the coexistence of traditional plant-based dietary patterns and modern, Westernised eating habits^(43,44)^. This variability may contribute to a more marked contrast across exposure groups, potentially allowing for a stronger detection of the benefits of a healthy lifestyle.

Our analysis of the individual components of the 2018 WCRF/AICR score revealed that the reduced risk of BC associated with high overall adherence was primarily driven by strong adherence to several key guidelines. Specifically, maintaining a healthy weight, being physically active, breast-feeding, consuming a diet rich in whole grains, vegetables and fruits and limiting the intake of processed foods high in fat, starches or sugars. Each of these recommendations was independently associated with a lower risk of BC in our study, a finding that was evident in both pre- and postmenopausal women. This aligns with the body of evidence demonstrating that healthy dietary and lifestyle patterns are protective against BC^(45)^.

The biological plausibility for this is well-established; diets abundant in plant-derived nutrients are rich sources of antioxidants, bioactive anti-inflammatory compounds and phytochemicals, which may mitigate cellular damage and inhibit disease progression^(46–48)^. Conversely, unhealthy dietary patterns, often energy-dense and nutrient-poor, are known to promote chronic inflammation, oxidative stress and hormonal imbalances, all of which are established risk factors for carcinogenesis^(45)^. Alongside diet, other lifestyle factors integral to the WCRF/AICR recommendations also play a crucial role. The global shift towards sedentary behaviours, a consequence of urbanisation and changing occupational patterns, has contributed to rising obesity rates and low physical activity levels in Morocco^(49)^. These lifestyle changes have significantly contributed to the emergence of NCD, including BC^(50)^. Regular physical activity is associated with cancer prevention through multiple biological mechanisms, including reducing insulin resistance, modulating inflammation and regulating hormone metabolism, all of which play a key role in carcinogenesis^(51)^. Likewise, maintaining a healthy weight is a well-established protective factor against BC, as excess body fat contributes to chronic inflammation and increased estrogen production^(52,53)^. Our findings also confirmed the protective role of breast-feeding, which likely reduces BC risk by limiting a woman’s cumulative lifetime exposure to sex hormones, such as oestrogen, through the induction of amenorrhoea and the differentiation of breast tissue^(54,55)^.

This study has several notable strengths. It is the first of its kind in Morocco and one of the few in an LMIC setting to investigate the association between adherence to the updated WCRF/AICR guidelines and BC risk. Furthermore, the large sample size and the use of a validated FFQ, which was adapted for the local context, allowed for a robust assessment of dietary intake. However, the findings should be interpreted in consideration of some limitations. Due to its observational nature, this case–control study may be susceptible to recall bias. To minimise this limitation, trained interviewers assisted participants during the interviews to ensure accurate and reliable responses to the questionnaire. Additionally, cases were recruited at the time of diagnosis and before the initiation of treatment to minimise potential recall bias and ensure that data on pre-diagnostic exposures were collected before any treatment-related stress or lifestyle changes could influence participants’ responses.

Furthermore, the choice of hospital-based controls, as previously discussed, may have introduced selection bias. While we attempted to minimise this by matching for age and place of residence, and by recruiting controls from among visitors of patients with non-related cancer disease, the potential for bias cannot be fully excluded. Also, it should be noted that the FFQ was not originally designed to assess the level of food processing of the different food groups, requiring assumptions to be made when classifying the food groups according to the Nova classification. Although residual confounding cannot be entirely ruled out, as risk factors are often interrelated, efforts were made to minimise this limitation by applying a comprehensive statistical adjustment to account for potential confounders that could influence both exposure and outcomes.

### Conclusion

In summary, the findings of this large-scale case–control study suggest that adherence to the 2018 WCRF/AICR guidelines is associated with a reduced risk of BC in Morocco, reinforcing the importance of comprehensive lifestyle modifications in BC prevention. Specifically, maintaining a healthy weight, engaging in regular physical activity, adopting a diet rich in plant-based foods while limiting processed foods and encouraging breast-feeding appear to be the key drivers of the observed inverse associations with BC. Future research should further explore the interaction between lifestyle factors and genetic predispositions to refine tailored prevention strategies for the Moroccan population.

## Table 1 Long description

The table compares the operationalisation of cancer prevention score components among cases and controls using the NCI standardised algorithm. It has 10 rows and 10 columns. The columns are labeled as follows: 2018 WCRF/AICR cancer prevention recommendations, Variables in the study, Operationalisation of the components of the score based on the NCI-led standardised scoring algorithm, Score, Controls (n 1373) n, Controls (n 1373) %, Cases (n 1373) n, Cases (n 1373) %, and P. The rows are labeled with different cancer prevention recommendations and their corresponding variables. Each row provides detailed data on the operationalisation of the components, including specific measurements and scores for both controls and cases. Notable trends and comparisons are highlighted across different categories such as BMI, waist circumference, physical activity, diet, and alcohol consumption.

Navigate back to Table 1.

## Table 2 Long description

The table compares various characteristics of the study population between case and control groups. It has 30 rows and 10 columns. The columns are labeled as Controls (1373), Cases (1373), and P value. The rows include Age, Age at menarche, Age at full-term pregnancy, Age at menopause, Wealth score, Moderate physical activity, BMI, WC, HC, Energy intake, Vegetables, Fruits, Unsaturated oils, Nuts and seeds, Whole grains, Red meat, Processed meat, Fibre intake, Area of residence, Marital status, Education level, Occupation, Oral contraceptive, Parity, Breast-feeding, Family history of cancer, Family history of breast cancer, Alcohol, and Smoking exposure. Each row provides mean, standard deviation, and P value for the respective characteristics. The table shows detailed data points for each characteristic, comparing the control and case groups.

Navigate back to Table 2.

## Table 3 Long description

The table presents data on the association between adherence to the 2018 WCRF/AICR cancer prevention recommendations and breast cancer risk. It includes columns for adherence score, controls (n and percent), cases (n and percent), unadjusted model (OR and 95 percent CI), and adjusted model (OR and 95 percent CI). The table is divided into three main sections: overall, premenopausal, and postmenopausal. Each section further breaks down adherence scores into three categories: less than or equal to 3.75, greater than 3.75 and less than or equal to 4.5, and greater than 4.5. Notable trends include a significant decrease in breast cancer risk with increasing adherence scores in both unadjusted and adjusted models across all sections. The P values for trends and one-point increments are all less than 0.001, indicating strong statistical significance.

Navigate back to Table 3.

## Table 4 Long description

.

Navigate back to Table 4.
